# Culture and Antimicrobial Consumption: Sector- and Class-Specific Differences in Country-Level Associations Across Europe

**DOI:** 10.3390/antibiotics15020186

**Published:** 2026-02-08

**Authors:** Timo J. Lajunen, Esma Gaygısız, Ümmügülsüm Gaygısız, Mark J. M. Sullman

**Affiliations:** 1 Department of Psychology, Norwegian University of Science and Technology, 7491 Trondheim, Norway; 2 Department of Psychology, University of Helsinki, 00014 Helsinki, Finland; 3 Department of Social Sciences, School of Humanities and Social Sciences, University of Nicosia, 1700 Nicosia, Cyprus; 4 Department of Economics, Middle East Technical University, 06800 Ankara, Turkey; 5 Department of Anesthesia Intensive Care, Faculty of Medicine, Gazi University, 06560 Ankara, Turkey

**Keywords:** antimicrobial consumption, antibiotics, Hofstede cultural dimensions, power distance, uncertainty avoidance, community vs. hospital, ECDC, correlation

## Abstract

Background: Antimicrobial consumption (AMC) varies widely across European countries, and cross-national studies have linked this variation to cultural values. However, two critical gaps remain: it is unclear whether these associations differ between community and hospital sectors or across antimicrobial classes. This study directly tests these differences. Methods: We analysed country-level AMC data from the European Centre for Disease Prevention and Control for EU/EEA countries, combining sector-specific (community, hospital) and Anatomical Therapeutic Chemical (ATC) group-specific data. Spearman’s rank correlation coefficients (ρ) were calculated between Hofstede’s cultural dimensions and AMC. We compared correlations across sectors within ATC groups, and between community antibacterials for systemic use (J01) and other community medicine classes, using differences in correlations (Δρ). Uncertainty was assessed with non-parametric bootstrap 95% confidence intervals and paired-label permutation tests, with false discovery rate control. Sensitivity analyses included leave-one-country-out checks and replication using Kendall’s τ-b. Results: Cultural values, especially Power Distance Index (PDI) and Uncertainty Avoidance Index (UAI), were more strongly associated with community antibiotic use than with hospital antibiotic use or other community medicine groups. PDI and UAI showed significantly stronger correlations with community J01 use than with hospital J01 use and with several other community ATC groups. These patterns were robust in sensitivity analyses. Conclusions: The national cultural context appears more closely related to community antibiotic use than to hospital use or other community medicines, particularly for PDI and UAI. This demonstrates that cultural drivers of AMC are context-specific, necessitating stewardship strategies tailored to community settings to address norms around hierarchy and uncertainty.

## 1. Introduction

Antimicrobial resistance (AMR) is a multifactorial public health threat, influenced by factors including infection prevention, healthcare access, and patterns of antimicrobial use. Antimicrobial consumption (AMC) varies widely across European countries and care sectors, with major implications for antimicrobial resistance and stewardship policy. Cross-national analyses indicate that these differences in AMC cannot be explained by clinical need alone; they are also shaped by socio-economic conditions, governance quality, and cultural values [1,2]. In particular, studies using Hofstede’s cultural framework [3,4] have linked higher Uncertainty Avoidance Index (UAI), Power Distance Index (PDI), and Masculinity (MAS) to greater national antibiotic use and to weaker infection-control performance indicators, suggesting that population-level norms and expectations influence prescribing and medicine-seeking behaviours [2,5,6].

Building on this work, Gaygısız et al. showed that cultural values account for a substantial share of between-country variation in antibiotic consumption when considered alongside socio-economic factors. UAI and MAS, and in some models PDI, were consistently associated with higher national antibiotic use, together with poorer governance and lower education levels [2]. The authors proposed that cultures characterised by strong aversion to ambiguity and action-oriented norms may favour “decisive” pharmacologic responses in primary care, reinforcing a tendency to prescribe antibiotics in situations of diagnostic uncertainty.

Evidence from infection control and resistance epidemiology supports this interpretation. Several studies have reported that European countries with higher UAI and PDI perform worse on stewardship-relevant indicators, such as prolonged surgical prophylaxis and higher methicillin-resistant Staphylococcus aureus (MRSA) prevalence, whereas low-UAI/low-PDI settings, such as Scandinavia and many Anglo-Saxon countries, achieve better outcomes [7,8]. These findings align with the view that antibiotic use and infection control are behavioural phenomena embedded in national culture and organisational practices.

Despite this growing literature, two important gaps remain. First, most cross-country analyses aggregate antibiotic consumption across healthcare sectors and do not formally contrast community and hospital use. Yet these settings differ in how cultural values are likely to exert their influence. Hospital prescribing is shaped by institutional protocols, specialist oversight, diagnostic infrastructure, and multidisciplinary stewardship programmes. By contrast, community prescribing is more exposed to patient expectations, pharmacy access, time pressure in primary care, and heterogeneous practice styles. If cultural values affect antibiotic use mainly through everyday expectations, perceptions of risk, and consultation dynamics, then stronger culture–use associations would be expected in community settings than in hospitals [1,5].

Second, few studies distinguish between different antimicrobial classes when examining culture–use relationships. Antibiotics are often treated as a single group, and it remains unclear whether culture is specifically coupled to community antibacterials for systemic use (ATC J01) or whether similar associations hold for other antimicrobial and anti-infective classes, such as antifungals, antivirals, and antimycobacterials. Clarifying this issue is important for interpretation: if cultural values correlate more strongly with J01 than with other community medicines, this would support the idea that culture shapes how clinicians and patients respond to acute symptomatic uncertainty in ways that are specific to antibiotics rather than to medicine consumption in general [2].

The present study addresses these gaps using country-level AMC data from the European Centre for Disease Prevention and Control (ECDC). We link Hofstede’s cultural dimensions to sector-specific (community, hospital) and class-specific (ATC groups) antimicrobial consumption and use a direct difference-in-correlations approach to compare the strength of culture–use associations within the same set of countries. This within-country comparison strategy allows us to focus on relative differences between sectors and classes, rather than on absolute correlation magnitudes considered in isolation.

Guided by this framework, we address two research questions.

**RQ1:**  *Are there differences in the strength of associations between Hofstede dimensions and medicine use across sectors, expressed as differences in Spearman correlations (Δρ = ρ[community] − ρ[hospital]) within ATC groups?*

**H1:**  *Culture–use associations will be stronger in the community than in hospitals for dimensions linked to decisiveness and hierarchy, particularly UAI and PDI, and to a lesser extent MAS, resulting in positive Δρ for these dimensions [2,5].*

**RQ2:**  *Are there differences in the strength of associations between Hofstede dimensions and community use of antibacterials for systemic use (J01) and other community medicine groups, expressed as Δρ = ρ[J01 community] − ρ[other community group]?*

**H2:**  *Cultural values, especially UAI and PDI, will be more strongly associated with community J01 consumption than with other community medicine classes, consistent with lower tolerance for ambiguity and stronger authority-driven expectations in antibiotic-seeking contexts [1,2].*

## 2. Results

To our knowledge, prior cross-national research has linked cultural dimensions to antibiotic consumption, but has not formally tested whether these culture–consumption associations differ between community and hospital sectors (or between antibiotic classes) using dependent-correlation contrasts. We therefore focus on statistically comparing correlation differences (difference in correlations, Δρ) across sectors and classes.

Analyses examined whether national cultural values, measured by Hofstede indices (Power Distance Index [PDI], Individualism [IDV], Masculinity [MAS], Uncertainty Avoidance [UAI], long-term orientation based on World Values Survey estimates [LTO/WVS], and Indulgence [IVR]), were differentially associated with medicine use in community versus hospital sectors, and whether culture was more strongly associated with community antibacterials (ATC J01) than with other community medicine groups. For each Hofstede dimension–outcome pair, Spearman’s rank correlation coefficient (ρ) was computed using pairwise complete observations to maximise the number of contributing countries.

To compare two dependent correlations sharing the same predictor (a Hofstede dimension) across two outcomes (for example, community vs. hospital, or community J01 vs. another community ATC group), the difference in correlations (Δρ) was calculated. Non-parametric 95% bootstrap confidence intervals (percentile method) were derived, together with a paired-label permutation test that swapped outcome labels within countries. The false discovery rate was controlled within each pre-specified family of comparisons. All sample sizes (N) reported in tables reflect the exact number of countries contributing data to each comparison.

### 2.1. Culture–Setting Comparisons (Hypothesis 1: Community vs. Hospital)

For each medicine group (antibacterials for systemic use [ATC J01], topical antifungals [D01BA], antimycotics [J02], antimycobacterials [J04], and antivirals [J05]), Spearman’s rank correlation coefficients (ρ) were first computed between each Hofstede dimension and consumption in the community and hospital sectors, respectively. The two dependent correlations were then compared by estimating the difference Δρ = ρ(community) − ρ(hospital), and testing whether Δρ differed from zero using paired-label permutation with non-parametric bootstrap confidence intervals.

**Figure 1:**
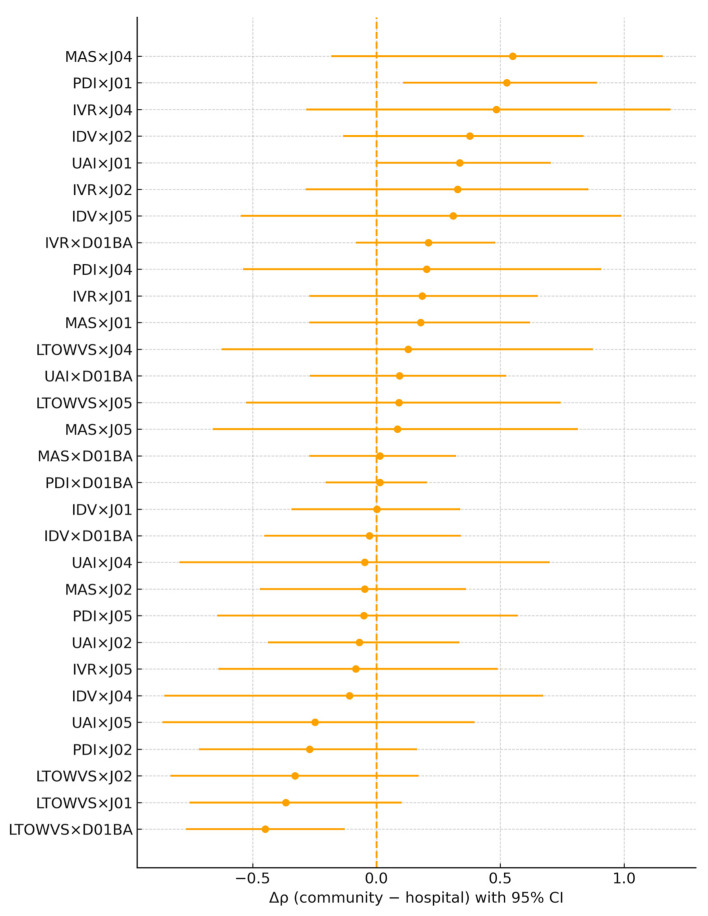
Differences in Spearman correlations (Δρ = ρ[community] − ρ[hospital]) between Hofstede dimensions and medicine use across ATC groups. Points show Δρ estimates with 95% bootstrap confidence intervals. The vertical dashed line denotes Δρ = 0. Positive values indicate a stronger culture–use association in the community than in hospitals.

As shown in Figure 1 and Table 1, two Hofstede–ATC contrasts reached statistical significance in the paired tests. For antibacterials (J01), PDI showed a stronger association in the community than in hospitals (Δρ = 0.53, 95% CI 0.11–0.89; paired-permutation *p* = 0.033; N = 25). In contrast, for topical antifungals (D01BA), LTO/WVS was more strongly associated in hospitals than in the community (Δρ = −0.45, 95% CI −0.77 to −0.13; *p* = 0.017; N = 22). Other Hofstede–ATC comparisons did not show statistically significant differences after paired testing. Overall, these findings are consistent with the hypothesis that cultural context exerts a stronger influence on community use for some medicine classes, particularly antibacterials.

### 2.2. Community Antibiotics Versus Other Community Medicine Groups (Hypothesis 2)

To assess whether culture was more strongly associated with community antibacterials (J01) than with other community medicines, Spearman correlations were compared for each Hofstede dimension between J01 community use and community use of J02, J04, J05, and D01BA, respectively. For each dimension, the difference in correlations was estimated as Δρ = ρ(J01 community) − ρ(other community group), with non-parametric bootstrap confidence intervals and paired-label permutation tests.

**Figure 2:**
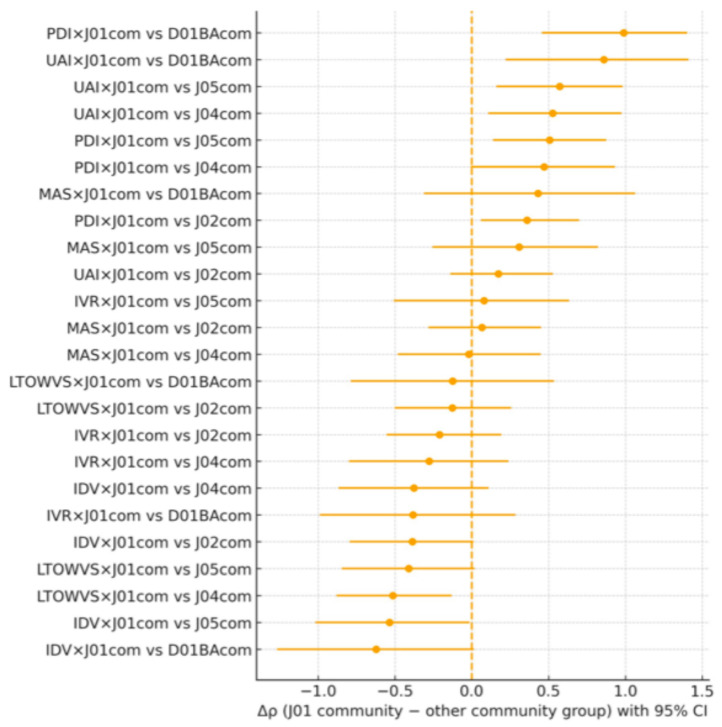
Differences in Spearman correlations in the community (Δρ = ρ[J01 community] − ρ[other community group]) between Hofstede dimensions and medicine use. Points show Δρ with 95% bootstrap confidence intervals. The vertical dashed line denotes Δρ = 0; positive values indicate stronger cultural association with community antibacterials (J01) than with the comparator community group.

As summarised in Table 2 and Figure 2, the most consistent signals were observed for PDI and UAI. For PDI, Δρ was positive and statistically significant when comparing J01 with D01BA (Δρ = 0.99, 95% CI 0.46–1.40; *p* = 0.005; N = 24), with J05 (Δρ = 0.51, 95% CI 0.14–0.88; *p* = 0.024; N = 25), and with J02 (Δρ = 0.36, 95% CI 0.06–0.70; *p* = 0.043; N = 25). For UAI, Δρ was positive and significant for comparisons of J01 with J05 (Δρ = 0.57, 95% CI 0.16–0.98; *p* = 0.024; N = 25), with D01BA (Δρ = 0.86, 95% CI 0.22–1.41; *p* = 0.030; N = 24), and with J04 (Δρ = 0.53, 95% CI 0.11–0.97; *p* = 0.031; N = 25). An exception was observed for LTO/WVS, where Δρ was negative for J01 versus J04 (Δρ = −0.51, 95% CI −0.88 to −0.13; *p* = 0.018; N = 26), indicating a relatively stronger association of LTO/WVS with J04 than with J01 in the community setting.

Taken together, these findings suggest that national cultural values, particularly PDI and UAI, are more strongly associated with community antibacterial use than with hospital antibacterial use or with several other community medicine classes. This pattern is consistent with the idea that cultural norms exert more direct influence in community settings. However, exceptions such as the LTO/WVS result for J04 indicate that cultural mechanisms may differ across therapeutic classes and warrant further investigation.

### 2.3. Sensitivity Analyses

Robustness was evaluated in four ways: (i) leave-one-out (LOO) deletion at the country level for Δρ; (ii) method checks using Kendall’s τ-b in place of Spearman’s ρ; (iii) comparison of permutation *p* values with bootstrap confidence intervals (already reported); and (iv) use of pairwise-complete observations to handle missingness and maximise N. For the significant culture–setting contrast in J01 (PDI) and the D01BA contrast (LTO/WVS) in Hypothesis 1, LOO analyses showed only small maximum absolute changes in Δρ and no reversals of sign. Similarly, for Hypothesis 2, the significant contrasts involving PDI and UAI retained direction and remained of comparable magnitude under LOO deletion. Kendall’s τ-b produced the same qualitative conclusions, in terms of both direction and relative strength across comparisons. Detailed LOO summaries for significant contrasts are provided in Table 3 and Table 4.

## 3. Discussion

The analyses suggest that national cultural values are systematically related to antibiotic use across European countries, and that these relationships vary by healthcare context. Power Distance Index (PDI) and Uncertainty Avoidance Index (UAI) showed stronger positive associations with community antibiotic consumption (ATC J01) than with hospital use. This pattern aligns with earlier European cross-national studies, which found that nations with higher PDI and UAI scores tend to have greater ambulatory antibiotic consumption and behaviours indicative of higher antibiotic demand [1,2,5]. The novel contribution of this work is to demonstrate that these culture-linked associations are significantly stronger in community care than in hospital settings. This indicates that the influence of cultural norms is more pronounced where diagnostic uncertainty and patient expectations are negotiated directly, compared to hospitals, where clinical protocols, specialist oversight, and microbiology support may buffer culturally shaped expectations and interaction norms.

The stronger association between PDI and community antibiotic use is consistent with a pathway in which hierarchical expectations and deference to authority shape both demand and prescribing. In higher power-distance settings, patients may be more likely to expect a decisive, tangible medical response from a trusted authority figure, and prescribers may experience stronger role expectations to act rather than to negotiate uncertainty, provide reassurance, or use delayed prescribing. Furthermore, in these high-PDI contexts, culturally shaped expectations for decisive action may be reinforced by medico-legal environments that incentivise defensive medicine. Prescribers, already positioned as authoritative figures, may perceive a greater risk of liability for perceived inaction, thereby amplifying the tendency towards tangible interventions such as antibiotic prescriptions. These tendencies echo prior interpretations that highlight how cultural values can structure consultation dynamics and the perceived legitimacy of requesting antibiotics [1,9]. The present results suggest that such dynamics are particularly influential where clinical encounters are frequent, time-limited, and directly visible to patients.

UAI also emerged as more strongly linked to community antibiotic use than to hospital use, supporting the view of antibiotics as an uncertainty-reducing response in routine care. Community presentations often involve non-specific symptoms, time pressure, and limited access to immediate diagnostic confirmation. In such contexts, both clinicians and patients may find reassurance in the perceived coverage offered by antibiotics, even when clinical benefit is unlikely. Borg (2012) framed UAI as a driver of inappropriate ambulatory antibiotic consumption [5], and a mixed synthesis of primary care studies likewise emphasised culturally embedded expectations, risk perceptions, and norms around managing uncertainty [9]. The current findings add that the association with UAI appears weaker in hospital settings, where objective testing and organisational controls are more routine. Consequently, our findings suggest that stewardship interventions designed to reduce diagnostic uncertainty, such as the implementation of rapid diagnostic testing (RDT), may be particularly effective in high-UAI cultural contexts. In these settings, RDT could directly address the core cultural driver of antibiotic demand by providing the tangible, objective evidence needed to offset the aversion to ambiguity, potentially yielding a greater reduction in inappropriate prescribing than in low-UAI settings where tolerance for uncertainty is higher.

The second hypothesis, that cultural values would relate more strongly to systemic antibacterials (J01) than to other medication groups, was also supported overall. The relative specificity of the culture–J01 associations suggests that the observed relationships are unlikely to be mere proxies for a general propensity to consume medicines or for broad national differences in access and utilisation. Instead, antibiotics appear particularly sensitive to culturally shaped norms around risk, reassurance, and what counts as legitimate medical action [1,5]. This interpretation is reinforced by evidence that culture can also predict how risk is managed through antibiotic choice: across Europe, UAI has been associated with broad-spectrum antibiotic use in community care [10].

Furthermore, the cultural imperative to reduce uncertainty may influence not only the volume but also the quality of antibiotic prescribing. Although our analysis at the ATC J01 group level did not distinguish between broad- and narrow-spectrum agents, prior European research indicates that higher national UAI is associated with greater community use of broad-spectrum antibiotics [10]. This suggests that in high-UAI settings, the aversion to diagnostic risk may drive prescribers towards antibiotics perceived as more empirically “reliable” or comprehensive, even when narrower-spectrum alternatives are clinically appropriate. Investigating this relationship with more granular consumption data represents an important future direction to understand how culture shapes therapeutic choices at the spectrum level. Cultural values are also likely to interact with institutional characteristics. Governance quality and corruption have been linked to antibiotic consumption and related national patterns, suggesting additional pathways through enforcement, informal practices, and trust in institutions [2,11].

The exceptions are theoretically informative. Long-term orientation (LTO) showed weaker associations with J01 than with several other medication groups, which may reflect the acute and episodic nature of many antibiotic decisions compared with medicines used for prevention or long-term condition management. LTO is commonly interpreted as reflecting planning, delay of gratification, and investment in future outcomes. These features plausibly map more naturally onto sustained pharmacotherapy and preventive behaviour than onto short-term decisions taken under symptomatic uncertainty. From this perspective, the LTO pattern provides a useful contrast, underscoring that cultural dimensions are not expected to align uniformly with all forms of medicine use.

Taken together, these patterns point to a plausible multilevel mechanism in which country-level cultural values shape individual expectations and clinician–patient interaction norms, which in turn influence the default response to symptomatic uncertainty in community care. Culture may also operate through healthcare safety culture and infection control norms, affecting how strongly guidelines are implemented and how deviations are sanctioned [6]. Complementing this, Borg and colleagues (2012) argued that European differences in methicillin-resistant Staphylococcus aureus (MRSA) epidemiology may reflect not only microbiology and resources but also deeper sociocultural and organisational determinants, including attitudes to compliance and risk [5]. Such work supports the broader inference that culturally embedded norms can influence both antibiotic consumption and the wider ecology in which resistance emerges.

While the present data are restricted to EU/EEA countries, it is important to consider how similar mechanisms linking culture and consumption may be observed in other health system contexts. In sub-Saharan Africa, factors such as limited access to prescribers and diagnostics, higher out-of-pocket costs, and a greater reliance on informal sources of medicine are likely to increase dependence on community-level purchasing. As a result, associations between cultural factors, including uncertainty avoidance and expectations related to hierarchy (power distance), may be more pronounced in relation to outpatient antibacterial consumption. In contrast, in some Latin American countries, stricter regulation of pharmacy dispensing and more consolidated public-sector healthcare pathways may result in a larger proportion of antibiotic use occurring within formal care. This is expected to reduce non-prescription access and may change the influence of cultural norms on both demand and prescribing. There is considerable heterogeneity within both regions, and similar cultural predispositions may lead to different patterns of consumption depending on the level of enforcement, affordability barriers, and the presence of stewardship infrastructure. Comparable context-dependence is also likely in parts of Asia. A global comparison, however, is outside the scope of this discussion.

The implications for antimicrobial stewardship and public health communication are straightforward but non-trivial. Uniform, one-size-fits-all approaches may underperform if they ignore culture-linked drivers. In higher power-distance contexts, stewardship strategies may benefit from leveraging credible authority and clear institutional endorsement, while simultaneously supporting clinicians with tools and organisational backing to refuse inappropriate requests without jeopardising trust [5]. In higher uncertainty-avoidant contexts, interventions that reduce diagnostic ambiguity or strengthen safety-netting may be particularly important, for example, rapid diagnostic testing where appropriate and clear follow-up plans, thereby reducing the appeal of just-in-case prescribing [9]. Across contexts, aligning messages and interventions with prevailing norms and institutional realities may help move beyond short-lived improvements towards more sustained change. These interpretations should be viewed in light of the study’s limitations and the ecological nature of the data, which are outlined in the following section.

### 3.1. Limitations

This study has several limitations that should temper interpretation. First, the country sample is relatively small and the analysis is ecological; associations observed at the national level cannot be assumed to reflect individual-level mechanisms, and unmeasured structural, epidemiological, or regulatory factors may confound some relationships.

Second, the measures rely on aggregated indicators. ECDC antimicrobial consumption data and national Hofstede scores, although widely used, simplify within-country heterogeneity and sector-specific prescribing cultures. National cultural scores are treated as static, even though cultural profiles can shift over time, and they may not fully align with the period covered by the consumption data. Similarly, AMC indicators are sensitive to differences in surveillance quality, reporting practices, and reimbursement systems across countries, which may introduce measurement error.

Third, the dataset is restricted to high-income, industrialised European countries. This limits generalisability and excludes lower-income settings and the global South, where antibiotic access, informal markets, regulatory environments, and disease burden may produce different culture–use dynamics. The findings should therefore be viewed as characterising one region and income range, rather than as universal patterns.

Fourth, the design is cross-sectional at the country level and focuses on a selected set of ATC groups. The analyses cannot establish causality, temporal ordering, or the direction of influence between culture and antimicrobial use, and they do not rule out alternative explanations involving governance, health system organisation, or concurrent stewardship interventions. Although the false discovery rate was controlled and sensitivity analyses were conducted, they do not rule out the possible effects of factors which we did not measure.

Fifth, we used pairwise-complete observations to maximise statistical power, which resulted in varying samples across comparisons. To assess potential bias, we conducted a post hoc analysis comparing the Hofstede dimension scores (PDI, UAI, etc.) and a key economic indicator (GDP per capita) between countries included versus excluded for the two ATC groups with the most missing data (J05 and D01BA). Using Mann–Whitney U tests, we found no statistically significant differences (*p* > 0.10 for all dimensions and GDP per capita in both comparisons). While this suggests that missingness was not strongly patterned by the cultural or economic factors measured here, we cannot rule out bias from unmeasured country-level characteristics related to surveillance capacity or reporting practices.

Finally, we used pairwise complete observations to maximise the number of contributing countries for each comparison. This approach improves statistical power but yields varying samples across ATC groups and Hofstede dimensions, which may affect comparability. If missingness is systematically related to consumption patterns, surveillance capacity, or cultural characteristics, some estimates of association or differences in correlations could be biassed.

### 3.2. Future Directions

Future research would benefit from a broader empirical base that extends beyond high-income European settings. Including low-income and middle-income countries, as well as regions with large informal markets and variable regulation, would allow stronger tests of whether the culture–consumption patterns observed here generalise to contexts with different constraints on access, enforcement, and disease burden. Such work should incorporate richer indicators of health system structure, governance, regulatory strength, and antibiotic availability, so that cultural values can be evaluated alongside competing explanations rather than standing in for them.

Methodologically, designs that exploit repeated annual data offer clear promise. Time-series or panel analyses using ECDC and similar datasets could help distinguish relatively stable cultural influences from shorter-term shifts driven by policy, epidemiology, or stewardship campaigns, clarify temporal ordering, and test whether the impact of interventions depends on the cultural profile of a country or region. Natural experiments, such as the staggered introduction of stewardship programmes or reimbursement changes, could be used to examine whether similar policies yield different consumption trajectories under different cultural conditions.

Multi-level and mixed-method approaches are also needed to connect national indicators to the mechanisms implied by this study. Studies that combine country-level cultural indices with individual-level data on expectations, risk perceptions, prescribing decisions, and consultation dynamics could test more directly the uncertainty-reduction and authority-relations pathways suggested here. Embedding qualitative work and experimental or intervention components, for example, culturally tailored communication tools or decision aids in primary care, would help identify which aspects of culture are most relevant for practice and how they can be addressed in antimicrobial stewardship.

Finally, as a reviewer suggested, a valuable extension would be to correlate cultural dimensions with AMR outcome data, such as rates of MRSA or ESBL-producing Enterobacterales. Analysing whether cultural values predict not only consumption, but also microbiological resistance outcomes would provide a more complete picture of the cultural epidemiology of AMR and help establish whether high-consumption, high-PDI/high-UAI countries also bear a disproportionate burden of resistance, further underscoring the public health urgency of culturally informed stewardship.

## 4. Materials and Methods

### 4.1. Data Source

Country-level antimicrobial consumption (AMC) data were obtained from the European Centre for Disease Prevention and Control (ECDC) Antimicrobial Consumption (AMC2) dashboard (https://qap.ecdc.europa.eu/public/extensions/AMC2_Dashboard/AMC2_Dashboard.html#eu-consumption-tab accessed on 3 September 2025). The dashboard compiles sector-specific community and hospital AMC metrics reported by European Union/European Economic Area (EU/EEA) countries to the European Surveillance of Antimicrobial Consumption Network (ESAC-Net), harmonised using the World Health Organization (WHO) Anatomical Therapeutic Chemical/Defined Daily Dose (ATC/DDD) methodology.

For this study, we extracted calendar year 2024 data, the most recent year available in the dashboard at the time of data retrieval. Although it would have been possible to compute multi-year averages (e.g., across several pre- or post-pandemic years), we selected a single, clearly defined post-COVID-19 year to improve interpretability, given that our outcome set included indicators potentially influenced by pandemic-related dynamics (e.g., antiviral use alongside antibacterial use). For each country, we extracted sector-specific and ATC group-specific AMC values exactly as reported by ECDC, without re-scaling or unit conversion. Where both total and sector-specific values were displayed, sector-specific values were used for all analyses. Variable names and abbreviations follow an internal project codebook used for data management and analysis.

Hofstede’s cultural value scores were retrieved from the following internet page: https://geerthofstede.com/research-and-vsm/ (accessed on 3 September 2025).

### 4.2. Variables

All variables used in the analyses are listed in Table 5. Consumption variables are sector-specific and expressed using ECDC’s standardised ATC/DDD metrics as provided on the dashboard. Country identifiers follow ISO standards.

### 4.3. Statistical Framework

The primary analysis employed a difference-in-correlations approach to test two main hypotheses. For each hypothesis, Spearman’s rank correlation coefficient (ρ) was calculated between Hofstede’s cultural dimensions and the relevant antimicrobial consumption (AMC) metrics. The core analysis involved computing the difference between two dependent correlations sharing the same cultural predictor (Δρ)—for example, the correlation with community-sector use minus the correlation with hospital-sector use for the same antimicrobial class. Statistical inference for Δρ was based on non-parametric bootstrap 95% confidence intervals (percentile method) and paired-label permutation tests within countries, with false discovery rate (FDR) control applied to families of comparisons. Robustness was assessed via leave-one-country-out sensitivity analyses and replication using Kendall’s τ-b. A detailed presentation of the results, including specific contrasts and sample sizes, is provided in Section 2 (Results).

### 4.4. Ethics Statement

This study analysed publicly available, aggregated country-level data from the ECDC AMC2 dashboard; no individual-level information was used. In line with this, research ethics committee review, informed consent, and data protection approvals were not required. The reliability and completeness of the data depend on national reporting to ESAC-Net and on the application of WHO ATC/DDD standards by ECDC and WHO; no additional validation of primary data was undertaken by the authors.

## Figures and Tables

**Table 1:** Culture–setting differences in correlations (Δρ = ρ[community] − ρ[hospital]) by Hofstede dimension and ATC group.

Hofstede	ATC Group	N	ρ (Community)	ρ (Hospital)	Δρ (Com − Hosp)	95% CI (Low)	95% CI (High)	*p* (Paired Perm.)	q (FDR)
PDI	J01	25	0.55	0.02	0.53	0.11	0.89	0.03	0.12
PDI	D01BA	21	−0.47	−0.48	0.01	−0.21	0.2	0.89	1.45
PDI	J02	23	0.19	0.46	−0.27	−0.72	0.16	0.29	0.32
PDI	J04	24	0.13	−0.07	0.2	−0.54	0.91	0.6	0.79
PDI	J05	22	0.11	0.17	−0.05	−0.64	0.57	0.9	1.45
IDV	J01	25	−0.29	−0.29	0.0	−0.34	0.34	0.99	4.96
IDV	D01BA	21	0.37	0.4	−0.03	−0.45	0.34	0.91	1.45
IDV	J02	23	0.07	−0.31	0.38	−0.13	0.84	0.14	0.22
IDV	J04	24	0.02	0.13	−0.11	−0.86	0.67	0.77	0.79
IDV	J05	22	0.14	−0.17	0.31	−0.55	0.99	0.41	0.53
MAS	J01	25	0.23	0.05	0.18	−0.27	0.62	0.46	0.7
MAS	D01BA	21	−0.37	−0.38	0.01	−0.27	0.32	0.93	2.33
MAS	J02	23	0.17	0.22	−0.05	−0.47	0.36	0.83	1.45
MAS	J04	24	0.33	−0.22	0.55	−0.18	1.16	0.14	0.22
MAS	J05	22	−0.07	−0.16	0.08	−0.66	0.81	0.8	0.96
UAI	J01	25	0.55	0.22	0.34	−0.0	0.7	0.06	0.12
UAI	D01BA	21	−0.29	−0.38	0.09	−0.27	0.52	0.7	0.79
UAI	J02	23	0.44	0.51	−0.07	−0.44	0.33	0.71	0.79
UAI	J04	24	0.08	0.13	−0.05	−0.8	0.7	0.92	1.45
UAI	J05	22	0.07	0.32	−0.25	−0.86	0.4	0.47	0.7
LTOWVS	J01	26	−0.28	0.09	−0.37	−0.75	0.1	0.1	0.14
LTOWVS	D01BA	22	−0.2	0.25	−0.45	−0.77	−0.13	0.02	0.02
LTOWVS	J02	24	−0.16	0.17	−0.33	−0.83	0.17	0.23	0.24
LTOWVS	J04	25	0.21	0.08	0.13	−0.62	0.87	0.74	0.79
LTOWVS	J05	23	0.03	−0.06	0.09	−0.53	0.74	0.8	0.96
IVR	J01	27	−0.01	−0.19	0.18	−0.27	0.65	0.46	0.53
IVR	D01BA	22	0.44	0.23	0.21	−0.08	0.48	0.19	0.22
IVR	J02	25	0.19	−0.13	0.33	−0.29	0.86	0.3	0.32
IVR	J04	26	0.24	−0.25	0.48	−0.28	1.19	0.23	0.24
IVR	J05	24	−0.19	−0.11	−0.08	−0.64	0.49	0.79	0.79

Footnote: Spearman’s ρ computed with pairwise complete observations. Δρ tested via paired-label permutation within country; 95% CIs by nonparametric bootstrap (percentile). FDR = Benjamini–Hochberg within this family. N = contributing countries.

**Table 2:** Community antibiotics (J01) versus other community medicine groups: differences in correlations by Hofstede dimension.

Hofstede	Comparison	N	ρ (J01 Community)	ρ (Other Community)	Δρ (J01 − Other)	95% CI (Low)	95% CI (High)	*p* (Paired Perm.)	q (FDR)
PDI	J01com vs. J02com	25	0.55	0.19	0.36	0.06	0.7	0.04	0.1
PDI	J01com vs. J04com	25	0.55	0.08	0.47	−0.01	0.93	0.07	0.1
PDI	J01com vs. J05com	25	0.55	0.04	0.51	0.14	0.88	0.02	0.04
PDI	J01com vs. D01BAcom	24	0.52	−0.47	0.99	0.46	1.4	0.0	0.02
IDV	J01com vs. J02com	25	−0.29	0.1	−0.39	−0.8	0.01	0.09	0.27
IDV	J01com vs. J04com	25	−0.29	0.09	−0.38	−0.87	0.11	0.2	0.27
IDV	J01com vs. J05com	25	−0.29	0.25	−0.54	−1.02	−0.01	0.06	0.1
IDV	J01com vs. D01BAcom	24	−0.2	0.42	−0.62	−1.27	0.01	0.1	0.27
MAS	J01com vs. J02com	25	0.23	0.16	0.07	−0.28	0.45	0.75	0.81
MAS	J01com vs. J04com	25	0.23	0.25	−0.02	−0.48	0.45	0.95	2.27
MAS	J01com vs. J05com	25	0.23	−0.08	0.31	−0.25	0.82	0.29	0.36
MAS	J01com vs. D01BAcom	24	0.14	−0.29	0.43	−0.31	1.06	0.21	0.27
UAI	J01com vs. J02com	25	0.55	0.38	0.17	−0.14	0.53	0.33	0.4
UAI	J01com vs. J04com	25	0.55	0.03	0.53	0.11	0.97	0.03	0.05
UAI	J01com vs. J05com	25	0.55	−0.02	0.57	0.16	0.98	0.02	0.04
UAI	J01com vs. D01BAcom	24	0.53	−0.33	0.86	0.22	1.41	0.03	0.04
LTOWVS	J01com vs. J02com	26	−0.28	−0.15	−0.13	−0.5	0.26	0.53	0.75
LTOWVS	J01com vs. J04com	26	−0.28	0.24	−0.51	−0.88	−0.13	0.02	0.02
LTOWVS	J01com vs. J05com	26	−0.28	0.13	−0.41	−0.85	0.02	0.08	0.1
LTOWVS	J01com vs. D01BAcom	25	−0.25	−0.12	−0.12	−0.79	0.54	0.71	0.81
IVR	J01com vs. J02com	27	−0.01	0.2	−0.21	−0.56	0.19	0.31	0.36
IVR	J01com vs. J04com	27	−0.01	0.27	−0.28	−0.8	0.24	0.37	0.4
IVR	J01com vs. J05com	27	−0.01	−0.09	0.08	−0.51	0.63	0.78	0.81
IVR	J01com vs. D01BAcom	25	0.03	0.41	−0.38	−0.99	0.28	0.27	0.27

Footnote: Spearman’s ρ computed with pairwise complete observations for each comparison. Δρ tested via paired-label permutation within country; 95% CIs by nonparametric bootstrap (percentile). FDR = Benjamini–Hochberg within this family. N = contributing countries.

**Table 3:** Sensitivity (leave-one-out) for significant Hypothesis 1 contrasts.

Hofstede	ATC Group	N	Δρ (Paired)	95% CI	p_Perm	LOO Δρ	LOO Max |Δρ Change|	LOO Sign Flip?
PDI	J01	25	0.53	[0.11, 0.89]	0.033	0.53	0.1	No
LTOWVS	D01BA	22	−0.45	[−0.77, −0.13]	0.017	−0.45	0.12	No

Footnote: LOO Δρ is recomputed after removing one country at a time; “max |Δρ change|” is the largest absolute deviation from the full-sample Δρ.

**Table 4:** Sensitivity (leave-one-out) for significant Hypothesis 2 contrasts.

Hofstede	Comparison	N	Δρ (Paired)	95% CI	p_Perm	LOO Δρ	LOO Max |Δρ Change|	LOO Sign Flip?
PDI	J01com vs. J02com	25	0.36	[0.06, 0.7]	0.043	0.36	0.09	No
PDI	J01com vs. J05com	25	0.51	[0.14, 0.88]	0.024	0.51	0.07	No
PDI	J01com vs. D01BAcom	24	0.99	[0.46, 1.4]	0.005	0.99	0.11	No
UAI	J01com vs. J04com	25	0.53	[0.11, 0.97]	0.031	0.53	0.1	No
UAI	J01com vs. J05com	25	0.57	[0.16, 0.98]	0.024	0.57	0.12	No
UAI	J01com vs. D01BAcom	24	0.86	[0.22, 1.41]	0.03	0.86	0.18	No
LTOWVS	J01com vs. J04com	26	−0.51	[−0.88, −0.13]	0.018	−0.51	0.13	No

Footnote: As above; all results computed with pairwise-complete observations to maximise N.

**Table 5:** Variables included into study.

Variable	Abbreviation	Definition
Country	Country	Country name
Antibacterials (community)	J01com	Community-sector consumption of antibacterials for systemic use (ATC J01)
Antibacterials (hospital)	J01hosp	Hospital-sector consumption of antibacterials for systemic use (ATC J01)
Topical antifungals (community)	D01BAcom	Community-sector consumption of topical antifungals (ATC D01BA)
Topical antifungals (hospital)	D01BAhosp	Hospital-sector consumption of topical antifungals (ATC D01BA)
Antimycotics (community)	J02com	Community-sector consumption of antimycotics for systemic use (ATC J02)
Antimycotics (hospital)	J02hosp	Hospital-sector consumption of antimycotics for systemic use (ATC J02)
Antimycobacterials (community)	J04com	Community-sector consumption of antimycobacterials (ATC J04)
Antimycobacterials (hospital)	J04hosp	Hospital-sector consumption of antimycobacterials (ATC J04)
Antivirals (community)	J05com	Community-sector consumption of antivirals for systemic use (ATC J05)
Antivirals (hospital)	J05hosp	Hospital-sector consumption of antivirals for systemic use (ATC J05)
Power Distance Index	PDI	Hofstede cultural index: acceptance of hierarchy/unequal power distribution at the national level (country-level covariate when applicable).
Individualism	IDV	Hofstede cultural index: degree of individualism vs. collectivism (covariate).
Masculinity	MAS	Hofstede cultural index: preference for competition/achievement vs. care/cooperation (covariate).
Uncertainty Avoidance	UAI	Hofstede cultural index: tolerance for ambiguity/uncertainty (covariate).
Long-Term Orientation	LTO/WVS	Hofstede cultural index (Long-Term Orientation; WVS-based extension where applicable) (covariate).
Indulgence	IVR	Hofstede cultural index: gratification vs. restraint (covariate).

## Data Availability

These data were derived from the following resources available in the public domain: https://qap.ecdc.europa.eu/public/extensions/AMC2_Dashboard/AMC2_Dashboard.html#eu-consumption-tab and https://geerthofstede.com/research-and-vsm/dimension-data-matrix/ (accessed on 3 September 2025).
